# Genetic diversity assessment of cucumber landraces using molecular signatures

**DOI:** 10.1186/s12864-024-10958-z

**Published:** 2024-11-06

**Authors:** Muhammad Sarmad Iftikhar, Hafiza Masooma Naseer Cheema, Asif Ali Khan, Ian Henson DeLacy, Kaye Enid Basford

**Affiliations:** 1 Department of Plant Breeding and Genetics, University of Agriculture Faisalabad, Faisalabad, 38000 Pakistan; 2 School of Biosciences, The University of Melbourne, Parkville, 3052 Australia; 3 Department of Plant Breeding and Genetics, Muhammad Nawaz Sharif University of Agriculture Multan, Multan, 60000 Pakistan; 4 School of Agriculture and Food Sustainability, The University of Queensland, Brisbane, 4072 Australia

**Keywords:** Cucumber, *Cucumis sativus*, EST-SSR, Genetic diversity, Landraces

## Abstract

Genetic profiling of the biodiversity in cultivated crop plants is necessary to preserve important genes and utilize them in a breeding program. Cucumber is used as a model plant to study various characteristics of *Cucurbitaceae*. Its adaptation to a wide range of climatic conditions suggested analyzing the landraces. The present study was conducted to evaluate the differences, at the genetic level, among landraces spanning five continents. DNA extracted from fifty-six landraces selected from USDA germplasm bank to cover a global representative sample of world cucumber landraces was used for polymerase chain reaction using twenty-eight polymorphic expressed sequence tags simple sequence repeat (EST-SSR) markers. Twenty-eight EST-SSR markers covering all seven chromosomes yielded 98 bands with an average of 3.42 bands per marker. Polymorphic information content ranged from 0.00 (EC35) to 0.74 (EC17) with an average of 0.34. Six clusters provided an appropriate summary of the variation among the landraces, with the two largest groups including 32 (Asiatic) and 17 (European and American) landraces, respectively. Four small groups, three with two members, and one with one member (PI 525155-Egypt) were dissimilar to the two main groups. Landraces from the same region were often clustered together. Genetic similarity of the landraces was revealed by marker banding patterns. The locations of genetic diversity for cucumber landraces can be identified from this study.

## Key message

Cucumber landraces have a broad genetic base distributed geographically. Genetic relationships among cucumber landraces were detailed. The route of the spread of cucumber from its center of origin to the rest of the world was elicited. The study confirmed that polymorphic EST-SSR markers are a valuable tool for assessing the genetic diversity of crops.

## Introduction

Cucumber (*Cucumis sativus* L.) is economically the most important crop belonging to the family *Cucurbitaceae* which is a major group of flowering plants consisting of 130 genera and 960 species [1, 2]. Among vegetables, cucumber ranks fourth in the world for its cultivated area after onion, tomato, and cabbage [3]. The genus ‘Cucumis’ has two species i.e., *Cucumis sativus* and *Cucumis hystrix* (2n = 2x = 24) with the latter being a wild species found only in China (province Yunnan). A wild variety of cucumber with the same chromosome number is *Cucumis sativus* var. hardwikii is found in the foothills of India [4]. The cucumber was first domesticated in India, 3000 years ago, and was introduced to China in 100 BC [5, 6].

The genetic diversity of plants and their conservation is becoming important due to the eradication of important genes by successive cycles of selections for higher yield [7]. It is argued that genes present in the landraces are crucial for climate-resilient crop breeding [8]. Elimination of landraces due to rapid urbanization, increased population and shrinking of arable land is a big threat for efforts to secure food [9]. Plant scientists are convinced that the genetic diversity of cultivated crops should be preserved to cope with future global food needs. The first step towards diversity preservation is to assess the present genetic diversity in a specific crop [10]. It is rational to think that maximum genetic diversity may be found where a crop was first domesticated. In all regions other than the center of origin, landraces can be found as a rich source of diversity [11]. Landraces are cultivars or dynamic populations having a distinct identity, origin, lacking formal crop improvement, adapted and associated with the local and traditional farming system, and often being genetically diverse. So, these can be valuable for diversity assessment and studies about evolution [12, 13].

Genetic diversity in crop plants can be assessed by comparing them based on morphology, biochemical properties or genetic makeup. While traditional methods like morphological and biochemical assessments provide valuable information, they often lack the resolution and specificity offered by molecular markers. The use of genetic markers allows for a more precise and detailed understanding of genetic variation at the DNA level, enabling researchers to identify specific loci associated with important traits. This approach has become increasingly common because it provides a direct measure of genetic differences, often revealing polymorphisms that are not detectable by other methods [14, 15]. To explore the diversity of genetic material, there are a variety of different marker systems e.g. RAPD (random amplification of polymorphic DNA), AFLP (amplified fragment length polymorphism), RFLP (restriction fragment length polymorphism), SSR (simple sequence repeats), EST-SSR (expressed sequence tags simple sequence repeats) and microsatellites [16, 17]. EST-SSR markers are helpful to assess genetic diversity as they use the expressed regions of DNA and simple sequence repeats surrounding them [18]. Efficacy of any marker system depends upon the reproductivity, polymorphism, number of alleles, cost, and efficacy of marker technology [19, 20]. The discriminatory power of SSR (simple sequence repeats) with low cost, high throughput sample processing, and simplicity would suggest that EST-SSR would have potential application in diversity analysis [21, 22].

In the current research, 56 landraces were selected from the USDA germplasm bank to assess the geographic spread of genetic diversity present in cucumber landraces. Twenty-eight polymorphic EST-SSR markers, selected to cover all seven chromosomes, were used for EST-SSR analysis; recorded data on the 98 bands (amplified by these 28 markers). These were used to conduct an improved PA using optimized dendrograms and classification enhanced biplots [23] to document and visualize the patterns of genetic relatedness. This analysis gave insight into the genetic relatedness among, the distribution of genetic diversity among, and the route of spread of the landraces from the center of origin to all over the world [24–26].

## Materials and methods

### Plant material

Seeds of 56 cucumber landraces were obtained from the germplasm repository of the Agricultural Research Service of USDA (ARS-USDA) spanning five continents except for Australia and Antarctica as representative samples from each country. All the landraces with their accession number, local names, country of origin and coding for analysis are presented (Table 1). All landraces belong to the taxon *C. sativus* var. sativus.

**Table 1:** Cucumber (*Cucumis sativus* var. sativus) landraces obtained from the USDA germplasm repository with their accession number, local name and country of origin (blank spaces in column “Local Name” shows that local name was not available). Coding was determined by country of origin (two letter country code and number of accessions from that country)

Code	Accession No.	Local Name	Country of Origin
PR1	PI 206,043		Puerto Rico
BR1	PI 118,279	Pepino verde comprido	Brazil
CA1	PI 229,808	55–64	Canada
CA2	Ames 3949	G 18,172	Canada
MU1	PI 525,075	Concombre blanc	Mauritius
ET1	PI 193,497	Elsgruber Znainer	Ethiopia
EG1	PI 525,150	8	Egypt
EG2	PI 525,155	55	Egypt
EG3	PI 288,237	Balady	Egypt
EG4	PI 188,749	Baladi	Egypt
HU1	PI 288,995	Nemet Kigyo	Hungary
HU2	PI 507,876	2,701,000	Hungary
ES1	PI 512,641	V-C-93	Spain
ES2	Ames 13,356	Medio largo verde	Spain
ES3	PI 261,608	Pepino medio largo verde	Spain
BA1	PI 357,854	Vodjanska	Bosnia
MK1	PI 357,832	Kusa	Macedonia
MK2	PI 368,560	Vratnicka	Macedonia
MK3	PI 357,834	Zelena	Macedonia
PL1	PI 285,603	Delikates	Poland
MD1	PI 263,080	Tiraspolski rannii	Moldova
GR1	PI 212,059	Kalivia	Greece
UA1	PI 263,078	Nezhinsky 12	Ukraine
RS1	PI 379,280	Bela	Serbia
AF1	PI 212,599	13,112	Afghanistan
CN1	PI 618,898	Xiao Ba Ca	China
CN2	PI 391,571	Nanking thorny	China
CN3	PI 103,049	Kuai Huang Kua	China
IN1	PI 605,987	USM 410	India
IN2	PI 217,644	13,765	India
IN3	PI 197,085	11,762	India
IN4	PI 164,284	8920	India
IN5	PI 271,337	1744	India
JP1	PI 227,210	Aibai	Japan
IQ1	PI 177,364	9884	Iraq
KZ1	Ames 19,038	Ames 19,038	Kazakhstan
RU1	PI 392,292	Iziascnyj	Russia
LB1	PI 218,199	Chebab	Lebanon
OM1	PI 532,162	Ashebe	Oman
PH1	PI 188,807	Pepino	Philippines
SY1	PI 181,752	9748	Syria
KR1	PI 281,448	9	South Korea
NP1	PI 427,230		Nepal
TR1	PI 338,236	Ames 1208	Türkiye
TR2	PI 178,887	9677	Türkiye
TR3	PI 169,398	Dikenli	Türkiye
TR4	PI 171,601	Rus Cinsi	Türkiye
TR5	PI 176,524	9480	Türkiye
IR1	PI 222,244	1424	Iran
IR2	PI 137,844	Khiyar	Iran
IR3	PI 137,845	Khiyar	Iran
PK1	PI 163,216	8307	Pakistan
PK2	PI 269,482	650	Pakistan
PK3	PI 330,628	Kshira	Pakistan
TH1	PI 249,562	Teang-rhan	Thailand
MM1	PI 200,818	94	Myanmar

### DNA extraction

Five seeds of each accession were sown in plastic pots filled with peat moss at the glasshouse of the University of Agriculture Faisalabad, Pakistan. DNA was extracted from young leaf tissues by using CTAB extraction methodology [27, 28]. The quality of DNA was checked through gel electrophoresis and quantity was measured by spectrophotometer at 260 nm following the manufacturer’s protocol [29]. The final concentration of DNA was adjusted up to 25 ng/µL.

### Polymerase chain reaction (PCR)

Each polymerase chain reaction (PCR) comprised of a volume of 20 µL. Each reaction contained 10× PCR buffer (2 µL), 25 mM MgCl_2_ (1.6 µL), one unit per microliter Taq polymerase (0.4 µL), 10 mM dNTPs (0.4 µL), 10 µM forward primer (1 µL), 10 µM reverse primer (1 µL), 25 ng/uL DNA (3 µL) and d_3_H_2_O (10.6 µL). Twenty-eight previously reported polymorphic markers [30], selected to cover all seven chromosomes, were synthesized for the current study from the commercial provider. PCR amplification was conducted using the following thermal cycling conditions: an initial denaturation at 94 °C for 8 min, followed by 40 cycles of denaturation at 94 °C for 1 min, annealing at 56 °C for 1 min, and extension at 72 °C for 1 min. A final extension was performed at 72 °C for 10 min. The amplified products were subsequently stored at -20 °C until further analysis.

### Polyacrylamide gel electrophoresis (PAGE)

The amplified products were mixed with 5 µL of bromophenol loading dye and each reaction was then resolved on 8% native PAGE along with 100 bp DNA ladder. The gel was run for 3 hours at 300 volts, stained with ethidium bromide dye and was photographed using a gel documentation system. For single-locus evaluations of the EST-SSR data, all DNA fragments were scored as presence or absence of each of the 98 bands. Only consistent bright bands were scored, and negative results were confirmed with re-examination.

### Analytical procedures

Bands were scored as present (1) or absent (0) and a two-way table of 56 landraces by 98 bands was made. Band sizes were estimated by comparing the migration distance of the amplified PCR products against a standard molecular weight marker (DNA ladder) run on the same gel. The DNA ladder provided reference bands of known sizes, allowing for accurate calculation of the band sizes based on their relative positions on the gel. Scoring data were copied to GENALEX 6.0 for calculating heterozygosity of markers (H), number of alleles amplified (Na) and Shannon’s information index (I) [31]. For the calculation of polymorphic information content (PIC) [32], data were copied to DARwin 6.0 [33] and analysis was carried out using its PIC function (Table 2).

**Table 2:** EST-SSR marker names with their coding (by English letters followed by the band number) for classification, heterozygosity (H), number of alleles (na), Shannon’s information index (I), polymorphic information content (PIC) ^*^ and size of amplified bands

Marker name	Coding	H	Na^1^	I	PIC	Size of amplified bands (bp)
EC 11	A	0.98	4.00	1.28	0.64	115, 199, 240, 260, 350
EC 12	B	0.58	5.00	1.27	0.60	156, 220, 250, 400, 900
EC 13	C	0.85	6.00	0.97	0.46	244, 260, 265, 310, 350, 355
EC 15	D	0.75	5.00	1.07	0.52	267, 270, 300, 310, 320
EC 17	E	0.96	9.00	1.73	0.74	300, 310, 340, 350, 370, 380, 400, 425, 510
EC 18	F	0.13	3.00	0.27	0.12	218, 225, 300
EC 19	G	0.00	1.00	0.00	0.00	168
EC 20	H	0.00	1.00	0.00	0.00	181
EC 22	I	0.18	2.00	0.30	0.15	192, 225
EC 23	J	0.22	2.00	0.34	0.18	224, 240
EC 24	K	0.80	5.00	0.98	0.47	234, 240, 260, 265, 500, 510
EC 27	L	0.75	3.00	1.08	0.58	287, 295, 340, 345
EC 28	M	0.06	2.00	0.14	0.06	237, 315
EC 31	N	0.96	4.00	0.98	0.49	160, 164, 200, 210, 250, 280
EC 33	O	0.45	4.00	0.86	0.42	100, 133, 160, 170
EC 34	P	0.82	4.00	1.01	0.51	150, 170, 191, 240, 250
EC 35	Q	0.06	2.00	0.13	0.05	200, 350
EC 38	R	0.92	3.00	1.03	0.55	200, 229, 250
EC 39	S	0.00	2.00	0.62	0.33	155, 275
EC 41	T	0.72	2.00	0.67	0.36	234, 290
EC 47	U	0.69	3.00	0.84	0.43	200, 220, 245
EC 49	V	0.00	1.00	0.00	0.00	219, 250
EC 50	W	0.00	1.00	0.00	0.00	223, 250
EC 52	X	0.96	3.00	0.80	0.41	186, 190, 210
EC 54	Y	0.72	5.00	1.17	0.55	267, 330, 335, 340, 345
EC 55	Z	0.60	4.00	1.00	0.49	280, 300, 320, 340
EC 56	AB	0.12	3.00	0.66	0.34	162, 220
EC 57	AC	0.12	2.00	0.27	0.13	150, 249

^*^GENALEX 6.0 was used to calculate heterozygosity of markers (H), number of alleles amplified (Na) and Shannon’s information index (I) [31] while DARwin 6.0 was used to calculate polymorphic information content (PIC) [33]

^1^Na is less in some cases than the number of amplified bands due to lower frequency of a band

Pattern analysis, joint use of clustering and ordination [34], was used to investigate the relationship among the landraces based on the presence or absence of the bands. These techniques were carried out in RStudio (R 1.0.136) and the results were visualized graphically [35]. Data were neither centered nor scaled for the analysis as the aim was to look upon actual differences in landraces caused by the 98 different bands amplified by the 28 markers. The dissimilarity between two landraces *i* and *i’*, required for clustering, was calculated using the Squared Euclidean Distance ($\:{d}_{i{i}^{{\prime\:}}}^{2}$):

$$\:{d}_{i{i}^{{\prime\:}}}^{2}=\frac{1}{\sum\:_{j=1}^{m}{n}_{j}}\sum\:_{j=1}^{m}\sum\:_{k=1}^{{n}_{j}}{\left({o}_{ijk}-{o}_{{i}^{{\prime\:}}jk}\right)}^{2}$$

where, $\:{o}_{ijk}$ is the score for the $\:k$^*th*^ band on the $\:j$^*th*^ marker for the $\:i$^*th*^ landrace, $\:i=1\dots\:,L$ the number of landraces (here 56), $\:j=1,\dots\:,m$ the number of markers (here 28), and $\:k=1,\dots\:,{n}_{j}$ the number of bands for the *j*^*th*^ marker. The Gower complement of this distance measure is the simple matching coefficient (SMC) because the data consisted of 0 and 1 only [36]. It was calculated by taking the dissimilarity measure away from 1 [37] as follows:

$$\:{SMC}_{i{i}^{{\prime\:}}}=1-{d}_{i{i}^{{\prime\:}}}^{2}$$

The SMC enables the inclusion of non-polymorphic markers in calculating the relationship matrix among genotypes [38].

The dendrogram was calculated using the SED matrix and UPGMA (Unweighted Pair Group Method with Arithmetic mean) [24, 39, 40] as a clustering strategy for both landraces and bands. These dendrograms were then optimized by applying a seriation algorithm based on the traveling salesman problem [23], which ensures that entities are arranged so that similar items are adjacent at the base of the dendrogram, enhancing the readability of the similarity across the grouping. Due to seriation, landraces lying close to each other were the most genetically similar, and the bands that produced similar patterns across landraces were grouped together, effectively clustering landraces with similar genotypes. This ensures that those landraces most similar genetically were positioned close together at the base of the dendrogram. Similarly, those bands that produced a similar pattern across landraces were grouped together. The chosen groups in each dendrogram were allocated different colors using the package [41].

Principal components were calculated using the base functions in R. The first three components (PC1, PC2, PC3) were plotted against one another in three biplots. Landraces were represented as points (with designated names) while bands were represented as vectors (arrows), using the same colors which identified the groups in both dendrograms, hence producing classification enhanced biplots [23].

## Results

The 28 EST-SSR marker combinations amplified a total of 98 bands among which 92 were high quality and polymorphic across 56 accessions. Four (EC 19, EC 20, EC 49 and EC 50) of the 28 markers were unable to produce polymorphic bands and amplified six monomorphic bands. These 28 EST-SSR markers were distributed on all seven cucumber chromosomes.

The number of bands ranged from one to nine with EC 17 producing the maximum, while two of the 28 markers (EC 19 and EC 20) amplified one band each (Table 2). Band size ranged from 100 bp (base pairs) to 900 bp with the highest amplified by marker EC 12. Band size 224 bp was found most frequently, 55 times in different landraces, while some bands were unique in the population, i.e. 260 bp, 265 bp, 310 bp, 370 bp, 510 bp, and 300 bp. There were ten relatively common bands with frequency > 85% (EC 18_218_ (0.935), EC 19_168_ (1.00), EC 20_181_ (1.00), EC 22_192_ (0.909), EC 23_224_ (0.891), EC 28_237_ (0.969), EC 35_200_ (0.972), EC 49_219_ (1.00), EC 50_223_ (1.00) and EC 57_249_ (0.923)). Polymorphic information content ranged from 0.05 to 0.74 with the highest for EC 17 and the lowest for EC 35. Eight of the markers with PIC values > 0.50 (EC 11, EC 12, EC 15, EC 17, EC 27, EC 34, EC 38, EC 54) were classified as informative for landrace identification. Heterozygosity ranged from 0.06 (EC 28 and EC 35) to 0.98 (EC 11) with an average of 0.55. Shannon’s information index (I) ranged from 0.27 (EC 18, EC 57) to 1.73 (EC 17) with an average of 0.81 (Table 2).

**Fig. 1:**
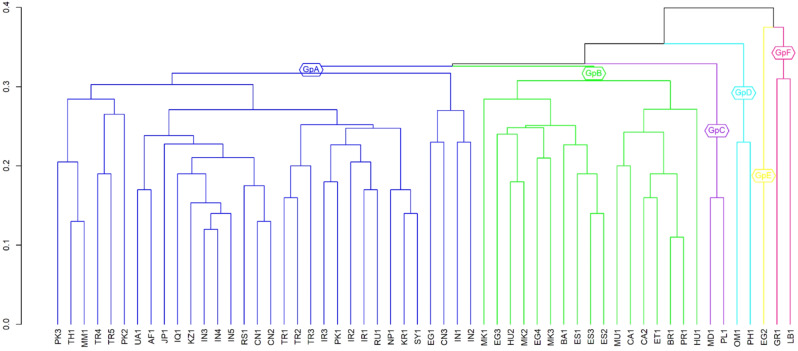
Optimized dendrogram for the classification of 56 cucumber landraces determined by the dissimilarity measure average squared Euclidean distance matrix calculated using the presence or absence among the 98 observed bands and the UPGMA clustering strategy. The labeling of landraces corresponds to the designation listed in Table 1

The optimized dendrogram for the landraces can be interpreted as a phylogenetic tree where landraces next to each other have a similar pattern of bands across the chromosomes and thus were more similar genetically. This was summarized at the six-group level (Fig. 1). At this level, there were two large groups and four small ones. The two large groups were denoted as Groups A and B, while others were denoted as Groups C to F (all in the order from left to right as they appear in the optimized dendrogram). Group A comprised 32 landraces, including all those from the Asiatic region, plus one from Egypt. Group B comprised 17 landraces from Europe, North and South America plus two from Egypt. The remaining Egyptian landrace (PI 525155) was in a single member-group, Group E. Group C had two landraces from Moldova (PI 263080) and Poland (PI 188807) from Europe, as did Group F from Greece (PI 212059) and Lebanon (PI 218199) (from near east), as did Group D from Oman (PI 532162) and the Philippines (PI 188807).

There was a major tendency for landraces from similar regions or countries to group together, indicating that they were similar genetically. The two landraces from South America, one from Puerto Rico and the other from Brazil were closely related and classified as one group as were the two from Canada, the three from Iran, and the three from Spain. The five Indian land races grouped into two groups of 3 and 2 members each, the five from Türkiye into two groups of 3 and 2 members, and the three from China into 2 and 1 member groups. The three landraces from Macedonia were closely related and grouped with a landrace from Hungary and with two of the landraces from Egypt. The exceptions were the landraces from Egypt, Greece and Hungary which were genetically dissimilar from each other.

**Fig. 2:**
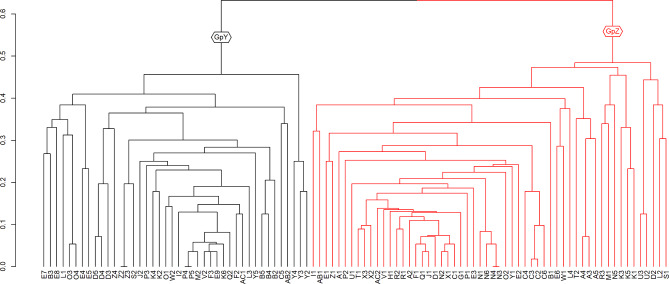
Optimized dendrogram for the classification of 98 bands determined by the dissimilarity measure average squared Euclidean distance matrix and the UPGMA clustering strategy. The labeling of bands corresponds to the designation listed in Table 1 (English letter followed by the number of band)

Another optimized dendrogram was constructed to visualize how the bands differed among the landraces (Fig. 2). Here we used a two-group summary, with forty-two in Group Y (on the left) and fifty-six in Group Z (on the right). All the bands produced by markers EC 11, EC 31, EC 38, EC 41, and EC 47 were grouped under the Group Z, while EC 12, EC 18, EC 19, EC 20, EC 28, EC 33, EC 35, EC 39, EC 49, EC 50, EC 54, EC 55, EC 56, and EC 57 had all its loci in Group Y except one (EC 249). The bands produced by other markers were scattered across both groups.

**Fig. 3:**
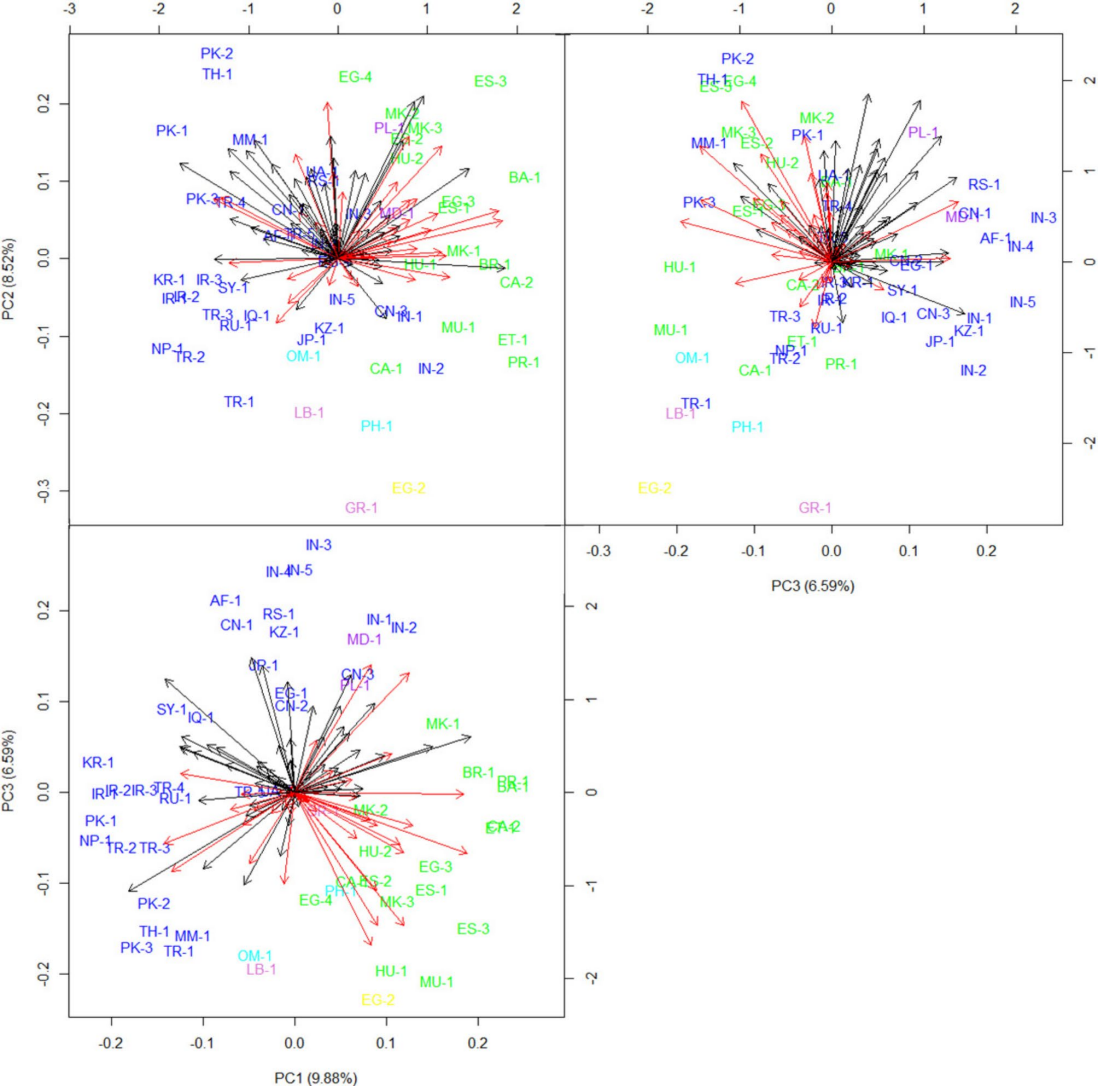
Classification enhanced biplot of first three principal components for 56 cucumber landraces and 98 bands (arrows) amplified using 28 EST-SSR markers. Color scheme is same as for classification of landraces and bands

The first three principal components from the ordination analysis accounted for 9.88%, 8.52% and 6.59% of the variation in the two-way (landrace by marker) array (Fig. 3). The first biplot (PC1 vs. PC2) presented the clear differentiation between the two large groups of landraces, Group A (blue) and Group B (green) (Fig. 3). The members of the four small groups were outliers in the biplot confirming that they are genetically different from other landraces.

**Fig. 4:**
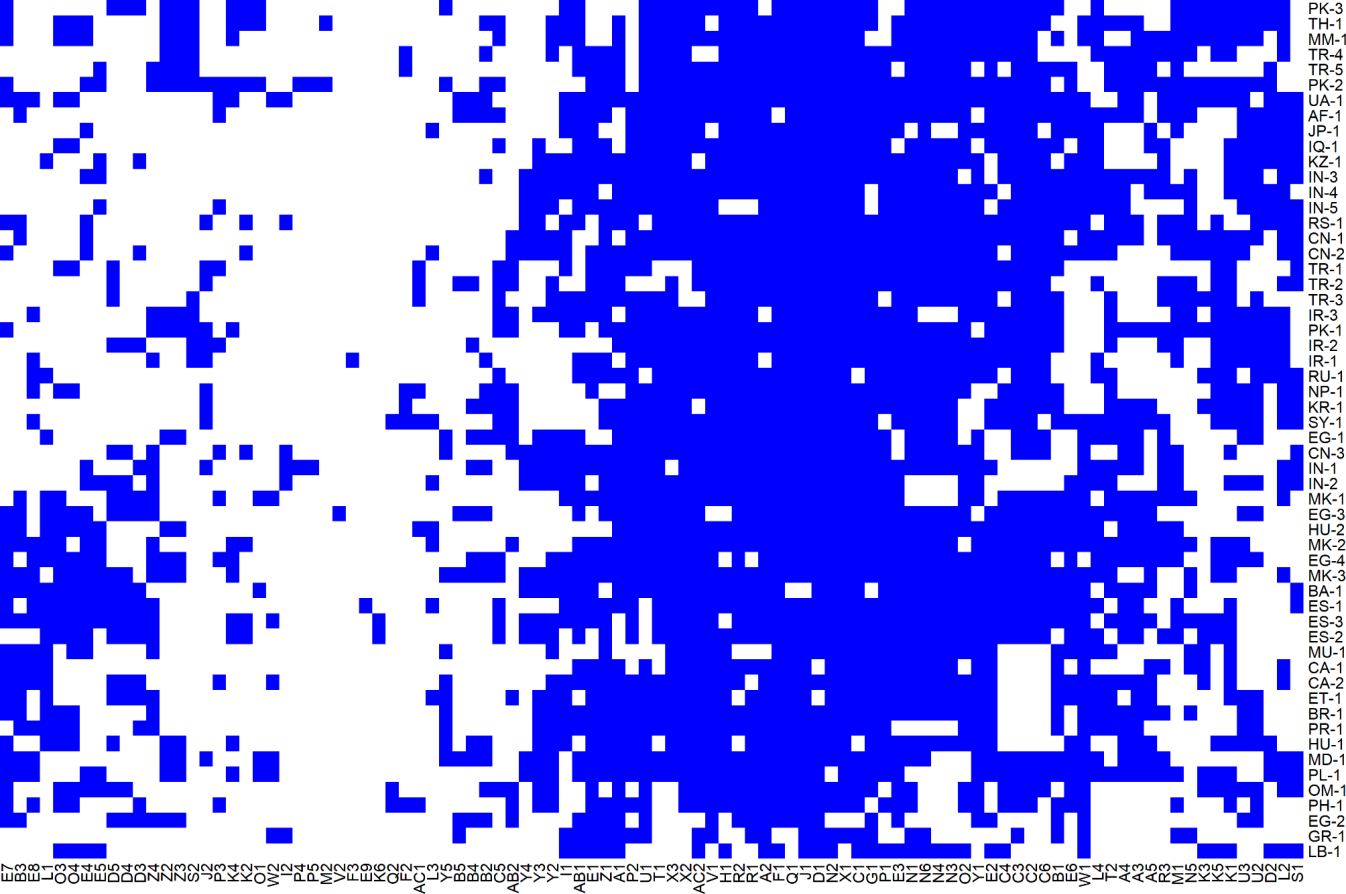
Population structure of 56 *Cucumis sativus* landraces depicted as a heatmap, where white color indicates the absence of a band and blue color indicates the presence of a band, based on 98 amplified bands. Landraces (on the vertical axis and coded as in Table 1) and bands (on the horizontal axis) are ordered according to their respective dendrograms

The population structure of the *Cucumis sativus* landraces was assessed using a heatmap representation of amplified marker bands, with the landraces and markers arranged according to their respective dendrogram orders (Fig. 4). The heatmap reveals distinct patterns of band presence and absence across the landraces, with clusters of landraces sharing similar banding patterns. These patterns suggest underlying genetic similarities among certain groups of landraces. Notably, a large portion of the bands (blue regions) are consistently present across multiple landraces, while other regions display more variability, indicating differential amplification of certain markers within the population.

## Discussion

The current study was designed to assess the genetic diversity among 56 cucumber landrace accessions representing all continents except Australia and Antarctica by using 28 polymorphic EST-SSR markers, selected from a previous study [30]. Four of these primers were monomorphic for our set of landraces contradictory to that previous study (where the primers were designed for specific lines) probably due to wider genetic base of landraces. It is suggested that eight of the remaining 24 markers should be used for landrace identification as they had PIC > 0.50 and were able to distinguish between the 56 landraces. In a previous study, 80 SSR markers were used to determine genetic diversity and the relationship of 35 Cucurbita rootstock accessions and showed that some of the markers were comprehensive to assess genetic variations [42]. Likewise, seventy SSR markers were used to assess genetic diversity in 44 cucumber accessions and it was found that they have wide genetic distance, which can be used for further exploration of cucumber landraces [43]. PIC, Shannon’s index, and heterozygosity suggested the importance of EST-SSR markers for use in genetic diversity assessment. Eight markers out of 28 were found highly informative and useful for the distinction among our set of landraces. This study reveals the usefulness of these eight markers as suggested in the previous study. These could be used for the same purpose for further work in cucumber. New EST-SSR markers were developed and used for genetic diversity assessment in various crops e.g. in sesame (*Sesamum indicum*); [44] and in hyacinth bean (*Lablab purpureus* L.) [45]. Mujaju also evaluated the watermelon germplasm of Zimbabwe and carried out the phylogenetic analysis and concluded that EST-SSR markers were good enough to discriminate between different types of melons [46]. Hence the use of SSR or EST-SSR is useful in assessing genetic diversity in cucumber and cultivar identification.

The investigation of the optimized dendrogram revealed that landraces from similar geographic areas were genetically alike, demonstrating the power of the seriation technique in highlighting fine differences among genotypes. This finding underscores the genetic similarities and differences among landraces from different regions and provides insights into the possible routes of cucumber spread from its center of origin in India to other parts of the world. Group A, being the largest, comprised landraces from Asiatic regions, suggesting that Asia possesses relatively similar genetic diversity in cucumber. However, none of the Pakistani landraces were like the Indian ones, which could be due to independent evolution in different countries. One Pakistani landrace (PI 163216, coded as PK1) was very similar to Iranian accessions (PI 137844, coded as IR2; PI 137845, coded as IR3), indicating a possible route of spread between these countries. Chinese landraces clustered with Russian and Turkish accessions, with one Chinese landrace (PI 103049, coded as CN3) clustering with an Indian one (PI 605987, coded as IN1), suggesting that cucumber spread from its primary center of origin (India) to the secondary center of origin (China). This study supports the thesis that the spread of cucumber followed a route from India to China, Russia, and then from China to Afghanistan, Iran, Türkiye, Iraq, and other Asian countries.

Group B comprised European landraces including from Spain, Macedonia, Bosnia and Hungry along with Canada, Brazil, and Mauritius. It can be hypothesized that cucumber was spread from the center of origin to Europe and the American region through the silk route. Group C comprised of landraces from Moldova and Poland suggesting that the genetic material in these countries is similar to each other and little different from other European accessions. Group D had two landraces from Oman (PI 532162, coded as OM1) and Philippines (PI 188807, coded as PH1). This could be due to the wider genetic base of Oman landraces. In another study, significant diversity was found in the landraces of Oman collected from four different regions [47]. It can be seen in the dendrogram that Egyptian landraces were spread in all the regions and one (PI 525155, coded as EG2) stood alone in Group E, suggesting the wider genetic base of Egyptian landraces. Group F had one landrace from Greece (PI 212059, coded as GR1) and one from Lebanon (PI 218199, coded as LB1).

All the landraces in Group A were from the Asiatic region suggesting that these have a narrow genetic base and similar to Indian germplasm. There was one exception that Egyptian landraces were scattered over groups A and B, possibly due to a broader genetic base and suggesting that Egyptian cucumber landraces originated from distant cucumber lines. It was found in a study of genetic diversity in African cucumber that Egyptian accessions are unique and have the potential to be used in breeding programs to broaden the genetic base [48]. Europeans have cucumbers different to those from the Asiatic region, so they were clustered separately from the Asiatic group. All but one of the European and the two South American landraces clustered together suggesting that they have genetic material in common. It was found in another study that Spanish landraces share alleles with the American region [49]. This report on landraces strengthens the hypothesis that the genetic stock of cucumber could have been transferred to America via Europe. However, the number of included landraces from these regions might not be enough to draw this conclusion. Groups C to F contained a few landraces and separated from their regions. It was possibly due to their broad genetic base and less genetic similarity with the landraces of the same region.

The heatmap highlights the genetic diversity within the cucumber landraces, as evidenced by their clustering based on their amplified marker profiles. The observed clustering corresponds with the dendrograms, indicating that the banding patterns are reflective of the genetic relationships among the landraces. The consistent presence of certain bands across numerous landraces may signify conserved genomic regions or markers associated with important traits, while the variability in other bands could point to regions under selective pressure or regions associated with environmental adaptation.

## Conclusion

The study was useful in indicating the route of spread of cucumber landraces from its center of origin to other places of the world. It was observed that Egyptian landraces may have broader genetic diversity and can be further explored for this purpose. The Asiatic region had almost similar landraces, while the American region got cucumber from Europe. We need to collect, explore, and exploit more germplasm to fulfill future needs of cucumber breeding with improved tolerance against biotic and abiotic stresses. The results from this study of cucumber landraces further showed the potential for the application of EST-SSR markers for genetic diversity assessment in cucumber.

## Data Availability

Data and materials are available upon request from the corresponding author.

## References

[1] Nunez-Palenius HG, Gomez-Lim M, Ochoa-Alejo N, Grumet R, Lester G, Cantliffe DJ. Melon fruits: genetic diversity, physiology, and biotechnology features. Crit Rev Biotechnol. 2008;28(1):13–55. 18322855 10.1080/07388550801891111

[2] Dhiman K, Gupta A, Sharma D, Gill N, Goyal A. A review on the medicinally important plants of the family Cucurbitaceae. Asian J Clin Nutr. 2012;4(1):16–26.

[3] FAO. Food and Agriculture Organization of the United Nations Rome, Italy: FAOSTAT. 2019 [cited 2021. https://www.fao.org/faostat/en/#data/QCL

[4] Prohens J, Nuez F. Handbook of plant breeding. Vegetables I: Asteraceae, Brassicaceae, Chenopodicaceae, and Cucurbitaceae. Valencia, Spain: Springer. Universidad Politecnica de Valencia; 2008.

[5] Bisognin DA. Origin and evolution of cultivated cucurbits. Ciência Rural. 2002;32:715–23.

[6] Qi J, Liu X, Shen D, Miao H, Xie B, Li X, et al. A genomic variation map provides insights into the genetic basis of cucumber domestication and diversity. Nat Genet. 2013;45(12):1510–5. 24141363 10.1038/ng.2801

[7] Zhou Z, Dong Y, Sun H, Yang A, Chen Z, Gao S, et al. Transcriptome sequencing of sea cucumber (Apostichopus japonicus) and the identification of gene-associated markers. Mol Ecol Resour. 2014;14(1):127–38. 23855518 10.1111/1755-0998.12147

[8] Munisse P, Bode S, Jensen BD. Diversity of landraces, agricultural practises and traditional uses of watermelon (Citrullus lanatus) in Mozambique. Afr J Plant Sci. 2011;5(2):75–86.

[9] Valcárcel JV, Peiró RM, Pérez-de-Castro A, Díez MJ. Morphological characterization of the cucumber (Cucumis sativus L.) collection of the COMAV’s Genebank. Genet Resour Crop Evol. 2018;65(4):1293–306.

[10] Govindaraj M, Vetriventhan M, Srinivasan M. Importance of genetic diversity assessment in crop plants and its recent advances: an overview of its analytical perspectives. Genet Res Int. 2015;2015(1):431487. 25874132 10.1155/2015/431487 PMC4383386

[11] Warburton M, Reif J, Frisch M, Bohn M, Bedoya C, Xia X, et al. Genetic diversity in CIMMYT nontemperate maize germplasm: landraces, open pollinated varieties, and inbred lines. Crop Sci. 2008;48(2):617–24.

[12] Brown AH. Genetic structure of crop landraces and the challenge to conserve them in situ on farms. Genes in the field: on-farm conservation of crop diversity. Ottawa, ON, CA: IDRC; 2000.

[13] Mercer KL, Perales HR. Evolutionary response of landraces to climate change in centers of crop diversity. Evol Appl. 2010;3(5–6):480–93. 25567941 10.1111/j.1752-4571.2010.00137.x PMC3352508

[14] Agarwal M, Shrivastava N, Padh H. Advances in molecular marker techniques and their applications in plant sciences. Plant Cell Rep. 2008;27(4):617–31. 18246355 10.1007/s00299-008-0507-z

[15] Davey JW, Hohenlohe PA, Etter PD, Boone JQ, Catchen JM, Blaxter ML. Genome-wide genetic marker discovery and genotyping using next-generation sequencing. Nat Rev Genet. 2011;12(7):499–510. 21681211 10.1038/nrg3012

[16] Nybom H. Comparison of different nuclear DNA markers for estimating intraspecific genetic diversity in plants. Mol Ecol. 2004;13(5):1143–55. 15078452 10.1111/j.1365-294X.2004.02141.x

[17] Kalia RK, Rai MK, Kalia S, Singh R, Dhawan A. Microsatellite markers: an overview of the recent progress in plants. Euphytica. 2011;177(3):309–34.

[18] Parthiban S, Govindaraj P, Senthilkumar SJB. Comparison of relative efficiency of genomic SSR and EST-SSR markers in estimating genetic diversity in sugarcane. 2018;8(3):144. 10.1007/s13205-018-1172-8 PMC5820232 29484283

[19] Varshney RK, Sigmund R, Börner A, Korzun V, Stein N, Sorrells ME, et al. Interspecific transferability and comparative mapping of barley EST-SSR markers in wheat, rye and rice. Plant Sci. 2005;168(1):195–202.

[20] Gong Y-m, Xu S-c, Mao W-h, Hu Q-z, Zhang G-w, Ding J, Li Y. -d. developing new SSR markers from ESTs of pea (Pisum sativum L). J Zhejiang Univ Sci B. 2010;11(9):702–7. 20803774 10.1631/jzus.B1000004 PMC2932880

[21] Tanksley SD, McCouch SR. Seed banks and molecular maps: unlocking genetic potential from the wild. Science. 1997;277(5329):1063–6. 9262467 10.1126/science.277.5329.1063

[22] Ramu P, Billot C, Rami J-F, Senthilvel S, Upadhyaya H, Reddy LA, Hash CT. Assessment of genetic diversity in the sorghum reference set using EST-SSR markers. Theor Appl Genet. 2013;126(8):2051–64. 23708149 10.1007/s00122-013-2117-6

[23] Arief V, DeLacy I, Basford K, Dieters M. Optimized dendrogram: extracting population information using seriation to enahance hierarchical clustering. 4th ICQG(Edinburgh). 2012.

[24] Arief VN, DeLacy I, Basford K, Dieters M. Application of a dendrogram seriation algorithm to extract pattern from plant breeding data. Euphytica. 2017;213:1–11.

[25] Arief VN, DeLacy IH, Payne T, Basford KE. Visualising the pattern of long-term genotype performance by leveraging a genomic prediction model. Australian New Z J Stat. 2022;64(2):297–312.

[26] Arief VN, DeLacy IH, Basford KE. Design and analysis of multi-year field trials for annual crops. Quantitative genetics, genomics and plant breeding. CABI Wallingford UK; 2020. pp. 178–93.

[27] Murray M, Thompson WF. Rapid isolation of high molecular weight plant DNA. NAR. 1980;8(19):4321–6. 7433111 10.1093/nar/8.19.4321 PMC324241

[28] Demeke T, Jenkins GR. Influence of DNA extraction methods, PCR inhibitors and quantification methods on real-time PCR assay of biotechnology-derived traits. Anal Bioanal Chem. 2010;396(6):1977–90. 19789856 10.1007/s00216-009-3150-9

[29] Ahn SJ, Costa J, Rettig Emanuel J. PicoGreen quantitation of DNA: effective evaluation of samples pre-or psost-PCR. Nucleic Acids Res. 1996;24(13):2623–5. 8692708 10.1093/nar/24.13.2623 PMC145983

[30] Hu J-b, Zhou X-y, Li J-w. Development of novel EST-SSR markers for cucumber (Cucumis sativus) and their transferability to related species. Sci Hort. 2010;125(3):534–8.

[31] Peakall R, Smouse PE. GENALEX 6: genetic analysis in Excel. Population genetic software for teaching and research. Mol Ecol Notes. 2006;6(1):288–95. 10.1093/bioinformatics/bts460 PMC3463245 22820204

[32] Bertolini E, Verelst W, Horner DS, Gianfranceschi L, Piccolo V, Inzé D, et al. Addressing the role of microRNAs in reprogramming leaf growth during drought stress in Brachypodium distachyon. Mol Plant. 2013;6(2):423–43. 23264558 10.1093/mp/sss160 PMC3603004

[33] Perrier X, Jacquemoud-Collet J. DARwin software. 2006.

[34] DeLacy I, Cooper M. Pattern analysis for the analysis of regional variety trials. Genotype-By-Environment Interaction and Plant Breeding Baton Rouge, LA, USA: Louisiana State University. 1990:301 – 34.

[35] Team R-S. R-Studio: integrated development for R. Boston, MA, USA: R-Studio, Inc.; 2015.

[36] Podani J. Extending Gower’s general coefficient of similarity to ordinal characters. Taxon. 1999;48(2):331–40.

[37] Podani JJT. Extending Gower’s general coefficient of similarity to ordinal characters. 1999;48(2):331 – 40.

[38] VanRaden PM. Efficient methods to compute genomic predictions. J Dairy Sci. 2008;91(11):4414–23. 18946147 10.3168/jds.2007-0980

[39] Podani J, Schmera D. On dendrogram-based measures of functional diversity. Oikos. 2006;115(1):179–85.

[40] Odong T, Van Heerwaarden J, Jansen J, van Hintum TJ, Van Eeuwijk F. Determination of genetic structure of germplasm collections: are traditional hierarchical clustering methods appropriate for molecular marker data? Theor Appl Genet. 2011;123:195–205. 21472410 10.1007/s00122-011-1576-x PMC3114091

[41] Galili TJB. Dendextend: an R package for visualizing, adjusting and comparing trees of hierarchical clustering. 2015;31(22):3718–20. 10.1093/bioinformatics/btv428 PMC4817050 26209431

[42] Kong Q, Chen J, Liu Y, Ma Y, Liu P, Wu S, et al. Genetic diversity of Cucurbita rootstock germplasm as assessed using simple sequence repeat markers. Sci Hort. 2014;175:150–5.

[43] Pandey S, Ansari WA, Mishra VK, Singh AK, Singh M. Genetic diversity in Indian cucumber based on microsatellite and morphological markers. Biochem Syst Ecol. 2013;51:19–27.

[44] Li-Bin W, Zhang H-Y, Zheng Y-Z, Wang-Zhen G, Zhang T-Z. Developing EST-derived microsatellites in sesame (Sesamum indicum L). Acta Agron Sinica. 2008;34(12):2077–84.

[45] Zhang G, Xu S, Mao W, Gong Y, Hu Q. Development of EST-SSR markers to study genetic diversity in hyacinth bean (Lablab purpureus L). Plant Omics. 2013;6(4):295–301.

[46] Mujaju C, Sehic J, Nybom H. Assessment of EST-SSR markers for evaluating genetic diversity in watermelon accessions from Zimbabwe. Am J Plant Sci. 2013;4:1448–56.

[47] Al-Rawahi M, Al-Said F, Khan I, Al-Khanjary S. Diversity of Cucumber accessions in Oman. Int J Agric Biology. 2011;13(4):505–10.

[48] Mliki A, Staub JE, Zhangyong S, Ghorbel A. Genetic diversity in African cucumber (Cucumis sativus L.) provides potential for germplasm enhancement. Genet Resour Crop Evol. 2003;50(5):461–8.

[49] Esteras C, Diez M, Pico B, Sifres A, Valcarcel J, Nuez F, Pitrat M, editors. Diversity of Spanish landraces of Cucumis sativus and Cucurbita ssp. Cucurbitaceae; 2008 21–24 May. Avignon (France).

